# Physicians’ satisfaction with providing buprenorphine treatment

**DOI:** 10.1186/s13722-019-0163-3

**Published:** 2019-08-26

**Authors:** Hannah K. Knudsen, Randy Brown, Nora Jacobson, Julie Horst, Jee-Seon Kim, Elizabeth Collier, Sanford Starr, Lynn M. Madden, Eric Haram, Alexander Toy, Todd Molfenter

**Affiliations:** 1 Department of Behavioral Science and Center on Drug and Alcohol Research, University of Kentucky, 845 Angliana Ave., Room 204, Lexington, KY 40508 USA; 2 Department of Family Medicine and Community Health, University of Wisconsin-Madison, 1100 Delaplaine Ct., Madison, WI 53715-1896 USA; 3 Institute for Clinical and Translational Research, University of Wisconsin-Madison, 4116 Signe Skott Cooper Hall, 701 Highland Ave., Madison, WI 53705 USA; 4 Department of Industrial and Systems Engineering, University of Wisconsin-Madison, 1513 University Ave., Madison, WI 53706 USA; 5 1067 Educational Sciences, 1025 West Johnson St., Madison, WI 53706-1706 USA; 6 1 West Wilson St. Rm 850, Madison, WI 53703 USA; 7 Ohio Department of Mental Health and Addiction Services, 30 E. Broad St., 8th Floor, Columbus, OH 43215 USA; 8 APT Foundation, 1 Long Wharf Drive, Suite 321, New Haven, CT 06511 USA; 9 Department of Internal Medicine, Yale University, New Haven, USA; 10 Haram Consulting, 413 River Road, Bowdoinham, ME 04008 USA

**Keywords:** Physician satisfaction, Buprenorphine, Opioid use disorder treatment

## Abstract

**Background:**

Buprenorphine is a critically important treatment for addressing the opioid epidemic, but there are virtually no studies of physicians’ job satisfaction with providing buprenorphine. Physicians’ job satisfaction has been linked to burnout and turnover as well as patients’ adherence to treatment recommendations, so it is important to understand how physicians’ satisfaction with providing buprenorphine treatment compares to their overall job satisfaction.

**Methods:**

As part of a cluster randomized clinical trial (RCT) focused on expanding access to medication for opioid use disorder, 55 physicians working in 38 organizations in Florida, Ohio, and Wisconsin completed a baseline web-based survey. Study measures included global job satisfaction, career satisfaction, and specialty satisfaction. Physicians who were waivered to prescribe buprenorphine were asked to rate their satisfaction with their current buprenorphine practice.

**Results:**

Overall, physicians were generally satisfied with their jobs, their careers, and their specialties. When waivered physicians (n = 40) were compared to non-waivered physicians (n = 15) on 13 satisfaction items, there were no statistically significant differences. Among waivered physicians, ratings for buprenorphine work were significantly lower than ratings for general medical practice for finding such work personally rewarding, being pleased with such work, and overall satisfaction.

**Conclusions:**

Although waivered and non-waivered physicians both reported high global job satisfaction, these data suggest that some waivered physicians may view their buprenorphine work as somewhat less satisfying than their global medical practice. Given that job dissatisfaction is a risk factor for turnover and burnout, managers of treatment organizations should consider whether strategies may be able to mitigate some sources of lower satisfaction in the context of buprenorphine treatment.

*Trial registration* ClinicalTrials.gov. NCT02926482. Date of registration: September 9, 2016. https://clinicaltrials.gov/ct2/show/NCT02926482

## Background

With more than 350,000 deaths in the US from 1999 to 2016 from opioids [1] and an additional 49,068 deaths in 2017 [2], the opioid epidemic has been declared a national emergency [3]. In part, opioid overdose deaths are the consequence of the lack of treatment of opioid use disorder (OUD) [4], a chronic medical condition that affects between 2.4 and 5 million Americans [5, 6].

Medication treatment using methadone, buprenorphine, or extended-release naltrexone is the gold standard of OUD treatment [7, 8]. Expanding medication treatment has been identified as a key strategy for addressing the opioid epidemic [9–11]. However, challenges to treatment expansion remain. Federal regulations and stigma surrounding methadone likely limited growth in licensed opioid treatment programs in the early years of the opioid epidemic [12], although some states have recently increased the number of opioid treatment programs (OTPs) or are planning such expansions [13]. Buprenorphine and naltrexone hold more promise for addressing the opioid epidemic because both can be prescribed in office-based settings, though they are more costly [14] and not always as effective as methadone [15]. The challenges of patient induction for extended-release naltrexone [16], coupled with its greater cost relative to buprenorphine [14], means that buprenorphine may have greater potential in expanding access to evidence-based treatment. Notably, buprenorphine has seen substantial growth in the numbers of patients and providers, particularly in medical offices outside the specialty treatment system [17–21].

Despite the growing number of providers who prescribe buprenorphine, relatively little is known about the satisfaction that physicians may derive from this aspect of their medical practice. More than a decade ago, Becker and Fiellin [22] conducted a systematic review and concluded that there was a gap in the literature about provider satisfaction with delivering buprenorphine treatment. Yet, there are virtually no studies of physicians’ satisfaction with providing buprenorphine published since their review, with the exception of a qualitative study of rural prescribers who found their buprenorphine practice to be rewarding and meaningful [23].

This limited research on physician satisfaction in the context of buprenorphine treatment is notable because the broader literature on physician satisfaction has identified a number of consequences of dissatisfaction. Although the majority of US physicians are satisfied [24], those who are dissatisfied are more likely to report burnout, intentions to leave their current jobs, and intentions to leave medicine entirely [25, 26]. Physician satisfaction also has implications for patient satisfaction [27, 28] and patient adherence to treatment recommendations [29].

The aims of the current study were to explore the satisfaction of physicians working in diverse OUD treatment settings. First, physicians holding the buprenorphine waiver were compared to physicians who were not waivered on measures of global job, career, and specialty satisfaction. Second, among the sub-sample of waivered physicians, their satisfaction with their buprenorphine practice was compared to their self-reported global job satisfaction.

## Methods

### Sample and data collection

As part of a cluster randomized clinical trial (RCT) focused on expanding access to medication for OUD (NCT02926482), 38 organizations in Florida, Ohio, and Wisconsin that were interested in expanding medication treatment were recruited for a 24-month study comparing two sets of implementation strategies [30]. The 38 organizations participating in the trial included 73 unique clinical sites. During the baseline period before deployment of the implementation strategies, invitations to participate in a web-based survey were distributed to physicians working within these organizations that provide treatment for OUD. The contact person for each organization participating in the RCT was asked to send the survey link to all physicians connected to the organization who were involved in treating patients with opioid use disorder. For those organizations that did not employ or contract with physician involved in treating patients with OUD at baseline, no surveys were distributed. On average, three reminders were sent to potential participants about completing the survey. Fifty-five physicians completed the survey, resulting in a response rate of 77%. All study procedures were reviewed and approved by the University of Wisconsin’s institutional review board.

### Measures

Items measuring physician satisfaction were drawn from the Physician Worklife Survey [31]. These 12 items asked physicians about their global job satisfaction (5 items), global career satisfaction about choosing to be a physician (4 items), and global specialty satisfaction (3 items). One additional item asked physicians about whether they felt adequately compensated. Response options ranged from 1 representing “strongly disagree” to 5 indicating “strongly agree”.

The five items measuring global job satisfaction from the Physician Worklife Survey were adapted to measure satisfaction with delivering buprenorphine treatment. These items asked physicians to rate their agreement with statements about finding their current buprenorphine clinical work rewarding, being pleased with their buprenorphine work, being satisfied with their current buprenorphine practice, reporting their buprenorphine work as a major source of frustration, and indicating their buprenorphine practice has not met their expectations. These items used the same response options as the overall satisfaction items.

Several physician characteristics were measured. Physicians indicated whether they were waivered to prescribe buprenorphine for OUD, and among those who were waivered, their current waiver type (i.e., 30 patients, 100 patients, or 275 patients). An open-ended question asked physicians to report their medical specialty, which was then coded into one of six mutually exclusive categories: (1) addiction (with no mention of any other specialty) (2) psychiatry (with no mention of any other specialty) (3) primary care (i.e., family medicine, internal medicine) (4) addiction and primary care (5) addiction and psychiatry, and (6) other. These categories were further collapsed into two groups: physicians who mentioned addiction in the open-ended question about specialty (e.g., addiction medicine, addiction and psychiatry, addiction and primary care; n = 27) and (2) all other physicians (e.g., only listed a primary care specialty, only listed psychiatry; n = 28). Physicians were asked if they were members of the American Society of Addiction Medicine (ASAM) and the American Academy of Addiction Psychiatry (AAAP). Demographic characteristics included age, gender, and race.

### Statistical analysis

Descriptive statistics were calculated to describe the characteristics of the sample. Given the small sample, this study relied upon t-tests and one-way analysis of variance (ANOVA) to examine physician satisfaction. Independent samples *t*-tests were used to compare waivered and non-waivered physicians on the measures of general satisfaction. Then, for the sub-sample of waivered physicians, paired sample *t-*tests were used to compare whether there were significant differences between general satisfaction and satisfaction with their buprenorphine practice. ANOVA was used to compare satisfaction with buprenorphine practice by waiver type, while independent samples *t*-tests were used to compare buprenorphine satisfaction of addiction specialists to all other physicians.

## Results

Of the 55 responding physicians, about 72.7% (n = 40) held the waiver to prescribe buprenorphine. Among those who were waivered, 10 physicians held the 30-patient waiver (25.0%), 14 physicians were waivered to treat up to 100 patients (35.0%), and 16 physicians held the 275-patient waiver (40.0%). The average age of the full sample was 53.3 (SD = 14.3), 68.5% (n = 37) were male, and the majority identified as white (70.9%, n = 39). The most prevalent specialties were addiction medicine (23.6%, n = 13), psychiatry (23.6%, n = 13), and primary care (23.6%, n = 13). About 16.4% (n = 9) identified as specializing in both addiction and primary care, 9.1% (n = 5) as specializing in both addiction and psychiatry, and 3.6% (n = 2) were categorized as other. More than half the sample were members of ASAM (54.6%, n = 30), but relatively few were members of AAAP (9.1%, n = 5). About 21.8% (n = 12) practiced in Florida, 41.8% (n = 23) in Ohio, and 36.4% (n = 20) in Wisconsin.

As seen in Table 1, physicians were generally satisfied with their jobs, their careers, and their specialties. Items with positive valences tended to have means near or above 4.0, indicating agreement, while items with negative valences (i.e., dissatisfaction) generally had means near 2.0, indicating disagreement. When waivered physicians were compared to non-waivered physicians on these 13 items measuring global satisfaction, there were no differences between the two groups. There were no differences in these measures of satisfaction by waiver type or between physicians who identified addiction as their specialty and those in non-addiction specialties (results not shown).

**Table 1:** Descriptive statistics of physician satisfaction (n = 55)

	Mean (SD)
*Global job satisfaction*	
I find my present clinical work personally rewarding	4.42 (0.57)
Overall, I am pleased with my work	4.36 (0.62)
Overall, I am satisfied with my current practice	4.16 (0.74)
My current work situation is a major source of frustration	2.41 (1.24)
My work in this practice has not met my expectations	2.04 (0.89)
*Global career satisfaction*	
If I were to choose over again, I would not become a physician	1.81 (1.17)
All things considered, I am satisfied with my career as a physician	4.22 (0.76)
In general, my medical career has met my expectations	3.98 (0.93)
I would recommend medicine to others as a career	3.73 (1.11)
*Global specialty satisfaction*	
My specialty no longer has the appeal to me it used to have	2.22 (1.29)
If I were to start my career over again, I would choose my current specialty	4.00 (1.15)
I would recommend my specialty to a student seeking advice	4.19 (0.97)
*Pay satisfaction*	
I feel adequately compensated for the medical services I provide	3.36 (1.21)

Items from the Physician Worklife Survey (Williams et al. [31]). Response options ranged from 1 = strongly disagree to 5 = strongly agree

Comparisons of global job satisfaction and buprenorphine-related satisfaction among the sub-sample of waivered physicians are presented in Table 2. There were three significant differences. Ratings for buprenorphine work were significantly lower than ratings for general medical practice with regard to finding such work personally rewarding, being pleased with such work, and overall satisfaction. However, physicians did not rate their frustration or unmet expectations differently for general medical practice versus buprenorphine-related work.

**Table 2:** Comparison of satisfaction with medical practice and with buprenorphine-related work among waivered physicians (n = 40)

	Medical practice	Buprenorphine work	*p-*value
	Mean (SD)	Mean (SD)	
Work is personally rewarding	4.46 (0.60)	4.08 (1.01)	0.023
Pleased with work	4.44 (0.64)	4.08 (0.90)	0.014
Satisfied	4.10 (0.82)	3.69 (1.03)	0.006
Work is major source of frustration	2.58 (1.31)	2.71 (1.21)	0.500
Work has not met expectations	2.05 (0.93)	2.24 (1.00)	0.164

Response options ranged from 1 = strongly disagree to 5 = strongly agree. Two-tailed paired sample *t*-tests

Buprenorphine-specific satisfaction was compared by waiver type, as seen in Table 3. There were no significant pairwise differences, after the Bonferroni correction, for the satisfaction items that were positively worded (i.e., rewarding, pleased, satisfied). There were significant differences regarding frustration and unmet expectations. Compared to physicians with the 30-patient waiver, physicians holding the 100-patient waiver more strongly endorsed buprenorphine work being a source of frustration. Physicians with the 100-patient waiver reported greater unmet expectations than physicians with the 30-patient waiver and physicians with the 275-patient waiver. Physicians who indicated addiction was their specialty (or one of their specialties) were compared to non-addiction physicians on these buprenorphine-specific measures, but there were no differences (results not shown).

**Table 3:** Satisfaction with buprenorphine-related work by waiver type (n = 40)

	30-patient waivered physicians	100-patient waivered physicians	275-patient waivered physicians	*p-*value
	Mean (SD)	Mean (SD)	Mean (SD)	
Buprenorphine work is personally rewarding	4.11 (0.78)	3.71 (1.20)	4.38 (0.89)	0.204
Pleased with buprenorphine work	4.33 (0.71)	3.79 (0.97)	4.19 (0.91)	0.303
Satisfied with buprenorphine work	4.00 (1.00)	3.14 (1.03)	4.00 (0.89)	0.040
Buprenorphine work is major source of frustration	1.89 (1.05)	3.43 (1.09)	2.50 (1.03)	0.005
Buprenorphine work has not met expectations	1.89 (0.78)	2.86 (1.17)	1.88 (0.62)	0.009

Response options ranged from 1 = strongly disagree to 5 = strongly agree. Analysis of variance (ANOVA) with p-value reported for the F-statistic

## Discussion

This study found physicians working in the sample of organizations providing substance use disorder (SUD) treatment were generally satisfied with their jobs, careers, and specialties. This is an important finding for the physician SUD workforce since poor physician satisfaction can impact intention to leave the profession and increase the likelihood of job turnover [32, 33]. Physician dissatisfaction can also negatively impact patients’ ratings of their care and no-show rates [34]. The ratings in our sample of SUD physicians for job satisfaction, career satisfaction, and specialty satisfaction scores were similar to scores from physicians in family medicine, internal medicine, and pediatric subspecialties in a previous study that used the same satisfaction instrument [35].

Among waivered physicians, their satisfaction with their overall medical practice was higher than some aspects of their buprenorphine practice. Although these differences were significant, perceptions of the rewarding nature and pleasure with both general medical practice and buprenorphine work still had mean scores above 4, indicating agreement in both cases. The biggest difference between general medical practice and buprenorphine work was for general satisfaction. The literature on barriers to delivering buprenorphine treatment, as reported by physicians, suggests that lack of psychosocial support, time constraints, limited peer and organizational buy-in, and lack of specialty back-up are among the largest concerns of physicians in prescribing buprenorphine [36–41], which may explain the lower general satisfaction. Having a greater understanding of the factors that contribute to physician dissatisfaction with buprenorphine work would assist organizations wanting to support physicians’ sustained acceptance of this pharmacotherapy; this is an important direction for future research. Of note, the negatively worded items (i.e., “major source of frustration” and “has not met expectations”) did not differ between general medical practice and buprenorphine work. However, those negatively worded items did differ by waiver type, with physicians holding the 100-patient waiver more strongly endorsing these negative attitudes. Future research should seek to elucidate the factors associated with these differences in satisfaction by waiver type.

Several limitations to the generalizability of these results should be considered. This is a very small sample that only includes prescribers from the states of Florida, Ohio, and Wisconsin. In addition, only organizations that were recruited into an RCT on pharmacotherapy capacity expansion were included in the study. The goal of the larger RCT likely impacted the uneven distribution of waivered and non-waivered physicians. Furthermore, this uneven distribution also likely reflects that the organizational contact was asked to forward the survey link to “physicians involved in treating opioid use disorder,” rather than all physicians in the organization. Reliance on an organizational contact for survey distribution also raises the possibility that not every physician treating patients with OUD received the survey. Response bias could have impacted the results (e.g. if non-response was greater among physicians less interested in or satisfied with medication treatments for OUD).

In addition, the sample was restricted to physicians. Buprenorphine prescribing was expanded to include nurse practitioners and physician assistants in 2016 and to clinical nurse specialists, certified registered nurse anesthetists, and certified nurse midwives in 2018. Understanding the satisfaction with buprenorphine work among these other types of medical professionals is an important direction for future research.

While concerns regarding generalizability do exist, the study highlights important directions for future research, such as comparing SUD physician satisfaction in their general medical practice to their buprenorphine work as well as examining potential similarities and differences in the factors associated with these two types of satisfaction. The findings should also be linked to the emerging body of research related to physician burnout [33] to determine the impact buprenorphine prescribing may or may not have on this issue.

## Conclusions

In the context of the opioid epidemic, there are ongoing concerns of shortages of buprenorphine prescribers [12, 42, 43] and behavioral health providers [44–46]. Physician satisfaction can be the driver of the use of evidence-based practices and physician retention [25]. A novel contribution of this research is to begin the study of the role of physician satisfaction in the delivery of SUD services. However, research is needed to identify the specific factors of buprenorphine treatment that contribute to physician dissatisfaction, as such information could inform interventions and other workplace changes that may reduce dissatisfaction. Future research should continue to study this issue and consider whether SUD physicians’ satisfaction is protective against burnout and beneficial in terms of clients’ outcomes.

## Data Availability

The datasets generated and analyzed for the current study are not publicly available to protect confidentiality of the participants, but are available from the corresponding author on reasonable request.

## Abbreviations

AAAP: American Academy of Addiction Psychiatry
ANOVA: analysis of variance
ASAM: American Society of Addiction Medicine
OUD: opioid use disorder
SUD: substance use disorder
RCT: randomized clinical trial
